# Unsupervised machine-learning classification of electrophysiologically active electrodes during human cognitive task performance

**DOI:** 10.1038/s41598-019-53925-5

**Published:** 2019-11-22

**Authors:** Krishnakant V. Saboo, Yogatheesan Varatharajah, Brent M. Berry, Vaclav Kremen, Michael R. Sperling, Kathryn A. Davis, Barbara C. Jobst, Robert E. Gross, Bradley Lega, Sameer A. Sheth, Gregory A. Worrell, Ravishankar K. Iyer, Michal T. Kucewicz

**Affiliations:** 1 University of Illinois, Dept. of Electrical and Computer Engineering, Urbana-Champaign, IL USA; 2 Mayo Clinic, Dept. of Neurology, Rochester, MN USA; 3 Mayo Clinic, Dept. of Physiology & Biomedical Engineering, Rochester, MN USA; 4 Gdansk University of Technology, Faculty of Electronics, Telecommunications and Informatics, Multimedia Systems Department, Gdansk, Poland; 5 Thomas Jefferson University Hospital, Dept. of Neurology, Philadelphia, PA USA; 6 University of Pennsylvania Hospital, Dept. of Neurology, Philadelphia, PA USA; 7 Dartmouth-Hitchcock Medical Center, Dept. of Neurology, Lebanon, NH USA; 8 Emory University, Dept. of Neurosurgery, Atlanta, GA USA; 9 UT Southwestern Medical Center, Dept. of Neurosurgery, Dallas, TX USA; 10 Baylor College of Medicine, Dept. of Neurosurgery, Houston, TX USA; 11 Czech Institute of Informatics, Robotics, and Cybernetics, Czech Technical University in Prague, Prague, Czech Republic

**Keywords:** Short-term memory, Biomedical engineering

## Abstract

Identification of active electrodes that record task-relevant neurophysiological activity is needed for clinical and industrial applications as well as for investigating brain functions. We developed an unsupervised, fully automated approach to classify active electrodes showing event-related intracranial EEG (iEEG) responses from 115 patients performing a free recall verbal memory task. Our approach employed new interpretable metrics that quantify spectral characteristics of the normalized iEEG signal based on power-in-band and synchrony measures. Unsupervised clustering of the metrics identified distinct sets of active electrodes across different subjects. In the total population of 11,869 electrodes, our method achieved 97% sensitivity and 92.9% specificity with the most efficient metric. We validated our results with anatomical localization revealing significantly greater distribution of active electrodes in brain regions that support verbal memory processing. We propose our machine-learning framework for objective and efficient classification and interpretation of electrophysiological signals of brain activities supporting memory and cognition.

## Introduction

Intracranial electroencephalography (iEEG) measures the electric field potentials in the brain using an array of grid, strip, and/or depth electrodes and enables the development of new diagnostic and treatment tools for neurological disorders^1,2^. Although all intracranially implanted electrodes broadly measure brain activity, the neurophysiological activity related to a specific cognitive task is observed in only a subset of electrodes^3–9^ located in the regions of brain associated with attention and the particular task. Identification of these “active” electrodes is important both for our understanding of the corresponding physiological processes and for developing better clinical treatments. The term ‘active’ refers here to signals sampled from brain regions electrophysiologically activated during a particular task rather than channels with active digitization of the signal. This type of active electrode classification in tasks requires utilizing the task-specific iEEG spectral signatures among the large-scale spectrum of brain activities^10–12^.

Selection of intracranial active electrodes has been most widely applied in epilepsy studies to find electrodes that are in the seizure onset zone^13–16^ by capturing the observable interictal and ictal pathological brain activity. Physiological brain activity has previously been used for the purpose of “channel selection” in motor-imagery tasks^17^, emotion tasks^18^, sleep stage detection^19,20^, and cognitive tasks^21^. The primary goal of these studies is to improve task-related classification performance, and techniques, which can be divided into two groups. The first set of techniques can be identified as *wrapper techniques*, which utilize “supervised learning” approaches to select subsets of electrodes based on task-related classification performance^17,22–24^. Typically, those techniques are computationally expensive and prone to overfitting^25^. The second set of techniques can be identified as *filtering techniques*, which utilize metrics based on spectral characteristics^26^, mutual information^21^, or signal statistics^18^ to obtain a subset of electrodes that satisfy some criteria chosen *a priori*. Other approaches include a combination of the above approaches and manual selection^18^. However, there are certain limitations of the above studies related to objectivity of the active electrode selection and interpretation of the physiologically relevant signals.

Most of the prior work on active electrode identification was demonstrated using scalp electroencephalography (EEG) recordings, in which the spatial arrangement of EEG electrodes was generally identical across subjects. Despite offering better coverage of the brain surface than the intracranial recordings do, they have lower spatial resolution, and cannot accurately measure high-frequency activity^27^ or target deep brain structures. Also, those studies have mainly focused on finding active electrodes for task-related classification problems and have not addressed the physiological relevance of the identified electrodes, leading to manual or semiautomatic identification of active electrodes in iEEG^3,4,9,28^. Studies aimed at addressing the physiological relevance of identified electrodes with automatic electrode selection have typically used a combination of different statistical methods based on iEEG power or event-related potentials^6–8^.

In this paper, we present a method that performs unsupervised classification of physiologically active iEEG electrodes in a fully automated fashion in three simple steps: it (1) takes normalized iEEG activity across different electrodes and subjects; (2) computes metrics derived from the spectrum of the normalized iEEG activity that quantify the power and synchrony of brain oscillations across a wide range of frequencies; and (3) uses the computed metrics to perform a binary machine-learning classification of active and inactive channels. We hypothesize that our method, which is based on simple classification of common spectral features, can identify a set of physiologically relevant electrodes to inform a study of brain functions. Our study is focused on the function of human verbal memory. Our method’s performance was measured against the results of a blinded, independent expert review who labeled active electrodes; the method was further validated by its demonstrated ability to locate active electrodes in brain regions that support verbal memory^29,30^.

## Methods

The data used in this study was collected from epilepsy patients who were implanted with iEEG electrodes while they performed verbal memory tasks. The data was preprocessed and converted to spectrograms to visualize its spectral content. Metrics that differentiate between electrodes based on spectral content indicative of cognitive processing were defined and extracted. To identify active electrodes, we performed unsupervised clustering using Gaussian Mixture Model (GMM) on the metrics. Finally, the performance of the metrics in identifying active electrodes was evaluated by comparing to ground-truth active electrodes. These steps are explained in more detail in the following subsections.

### Data collection and preprocessing

We used a large public dataset^31^ of intracranial recordings made during free recall memory tasks in patients undergoing iEEG monitoring as part of clinical treatment for drug-resistant epilepsy. Data were collected from the following clinical centers: Mayo Clinic, Thomas Jefferson University Hospital, Hospital of the University of Pennsylvania, Dartmouth-Hitchcock Medical Center, Emory University Hospital, University of Texas Southwestern Medical Center, and Columbia University Hospital. The research protocol was approved by the respective IRB at each clinical center, and informed consent was obtained from each participant. All methods were performed in accordance with the relevant guidelines and regulations approved by the respective IRB. Electrophysiological data were collected from electrodes implanted on the cortical surface and into the brain parenchyma. Electrophysiological data were collected from standard clinical subdural and penetrating depth electrodes (AdTech Inc., PMT Inc.) implanted on the cortical surface and into the brain parenchyma, respectively. The subdural electrode contacts were arranged either in a grid or a strip configuration with contacts separated by 10 mm. The depth electrode contacts were separated by 5–10 mm spacing^32^. For each subject, the number and placement of the electrodes were determined by a clinical team with the goal of localizing epileptogenic brain regions as part of the patient’s evaluation for epilepsy surgery.

Following electrode implantation, each subject participated in delayed free-recall memory tasks^33^, in which they studied lists of words presented on a laptop computer screen for a later memory test. Each list was composed of 12 words chosen randomly without replacement from a pool of common nouns. Words from the list were shown sequentially; each word remained on the screen for 1600 ms, followed by a randomly jittered 750–1000 ms blank inter-stimulus interval. Immediately following the final word in each list, participants performed a math distractor task (>20 s) consisting of simple arithmetic problems. Following the distractor task, participants were given 30 seconds to verbally recall as many words as possible from the list in any order. Each session consisted of 25 lists of this word presentation-distractor-recall procedure. Subjects who had at least one session were included in our study. iEEG data recorded from the electrodes were synchronized with the task computer through use of a TTL signal for timestamping events in the electrophysiological recordings.

A bipolar montage (derivative) was calculated *post hoc* for each subject, resampled to a common frequency of 500 Hz. Each electrode pair in the bipolar montage results in one signal derived from subtracting signals from a particular pair of contacts. Henceforth, each bipolar signal was defined as an electrode^3,4,32,34,35^. For any one subject a bipolar electrode was thus calculated by subtracting the measured voltage time series on all combinations of spatially adjacent contacts along the same row/column. This resulted in *N* − 1 electrodes in case of the penetrating depth and strip electrodes, and *N* = (*i* − *1*)  × *j* + (*j* − *1*) × *i* electrodes on the grid electrodes, where *i* and *j* are the numbers of contacts in the vertical and horizontal dimensions of the grid. Note that some of these bipolar electrodes will share the same electrode contacts and thus not all signals can be considered to be truly independent. There were 11,869 electrodes, i.e., bipolar pairs with 103 (32) electrodes per subject on average (standard deviation is provided in the parentheses.) Out of the 11,869 electrodes, 5186 were subdural and 6683 were depth, with each subject having 45 (51) subdural and 58 (51) depth electrodes on average.

Subjects had electrodes in multiple regions of the brain including temporal lobe (N = 106 subjects), parietal lobe (N = 96 subjects), frontal lobe (N = 107 subjects), limbic lobe (N = 109 subjects), occipital lobe (N = 58 subjects), and sub-lobar regions (N = 78 subjects). 91 subjects had electrodes in the right cerebrum while 98 had electrodes in the left cerebrum. All the sessions and electrodes were retained for further analysis.

### Analysis of spectral characteristics specific to active electrodes

**Figure 1:**
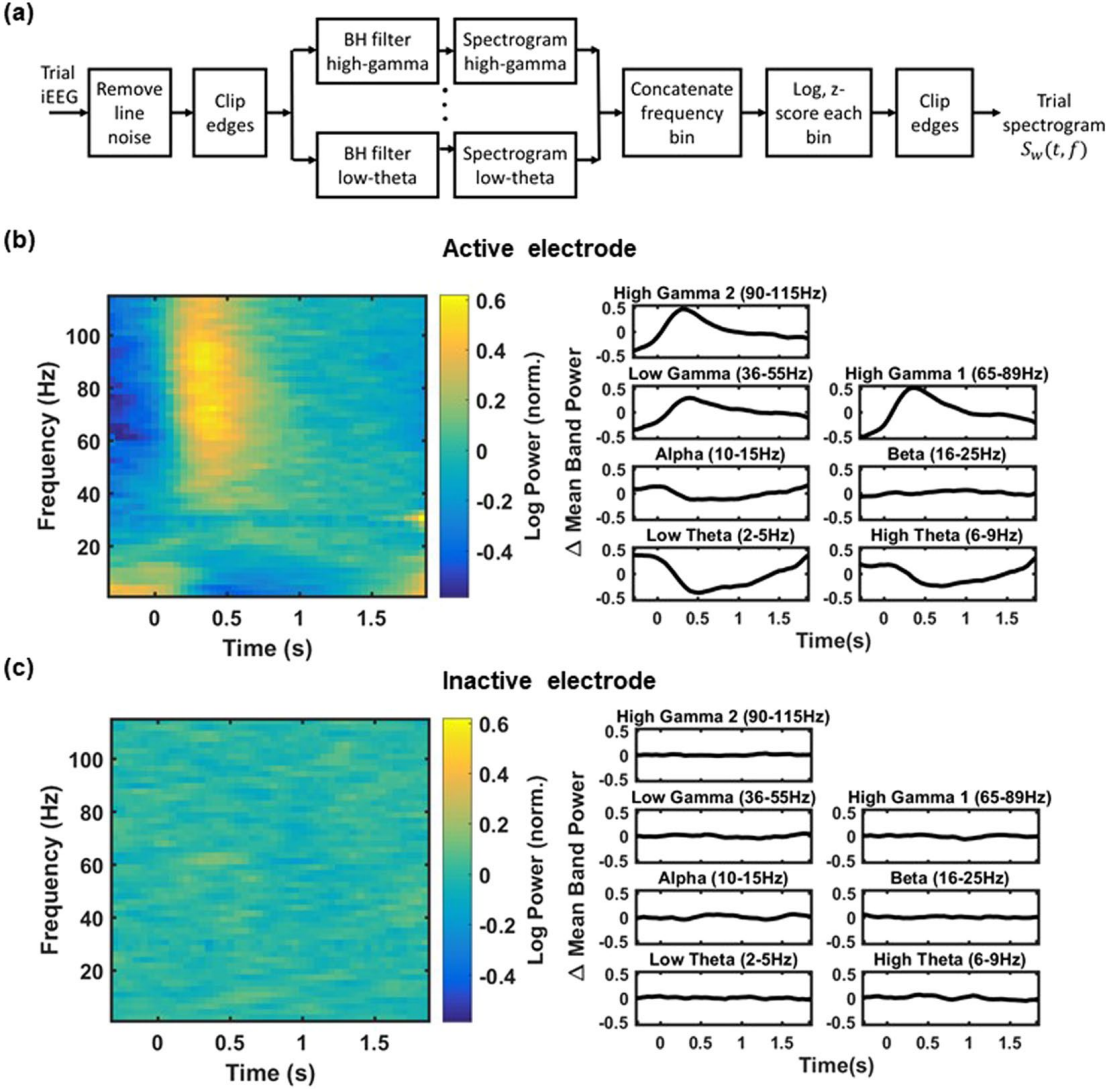
Spectrogram computation and trial-averaged power changes recorded from example active and inactive electrodes. (**a**) Schematic of the steps involved to obtain a trial’s spectrogram from its iEEG signal. In sub-figures (**b**,**c**), the left panels show trial-averaged spectrograms from all trials for an example subject. The right panels show the mean band power changes, i.e., power that was averaged within the defined frequency ranges *low theta* (2–5 Hz), *high theta* (6–9 Hz), *alpha* (10–15 Hz), *beta* (16–25 Hz), *low gamma* (36–55 Hz), *high gamma 1* (65–89 Hz), and *high gamma 2* (90–115 Hz). (**b**) The trial-averaged spectrogram and mean band power changes for an active electrode. Notice distinct power changes in the theta and gamma bands characteristic of stimulus processing. These trends are summarized in plots for the mean band power change in the corresponding frequency bands. (**c**) Trial-averaged spectrogram and mean band power change as in (**b**) but for an inactive electrode.

We first visualized the spectral content of the electrophysiological signals and then quantified it as spectrograms. The following data preprocessing was done in a subject-specific manner. The iEEG data were divided into epochs corresponding to each presented word. Each epoch consisted of 1600 ms of word viewing on the screen and a 700 ms inter-stimulus interval before and after the word presentation, resulting in a total epoch length of 3000 ms. An IIR notch filter at 60 Hz with 10 Hz bandwidth was used to remove line noise from all the recorded iEEG data. 100 ms of signal was removed from both ends to eliminate convolution edge artifacts. The truncated signal was filtered with Barlett-Hanning filters (1000 order) in the low-frequency bands *low theta* (2–5 Hz), *high theta* (6–9 Hz), *alpha* (10–15 Hz), and *beta* (16–25 Hz), and in the high-frequency bands *low gamma* (36–55 Hz) and *high gamma* (65–115 Hz). We band-pass filtered the truncated signal before estimating spectral power in the given bands to eliminate the effect of power from frequencies beyond a particular band of interest. This signal processing procedure was applied to minimize the power-law effect of lower frequencies on estimating power in the higher frequency bands. To convert the resulting signal to a spectrogram with a 2-Hz frequency bin resolution from 2 Hz–115 Hz, we used 500-ms sliding windows with 50-ms slide lengths, log-normalized and z-scored within the frequency bins to address the power law disparity between decreasing amplitude and increasing frequency. We used a mean and standard deviation calculated from the entire epoch of word encoding to normalize our signals with z-score transformation for each epoch of word presentation. This resulted in one spectrogram of power changes induced on a particular word presentation recorded on an electrode. To deal with any edge artifacts, we clipped two time points from either end of the spectrogram for all frequencies after the z-score normalization. For each electrode, we averaged the spectrograms across all word epochs to obtain trial-averaged spectrograms (Fig. 1). Figure 1a schematically shows the data pre-processing steps.

Let the spectral power in the spectrogram be denoted by

1 $$\begin{array}{c}{S}_{w}(t,f),w\in W,t\in T,f\in F,\end{array}$$

where *w* denotes trial (word), *t* denotes time, *f* denotes frequency, and *W*, *T* and *F* respectively denote the ranges of values w, *t* and *f* can take. The parameters for the zscore normalization – mean *μ*_*w*_(*f*) and standard deviation *σ*_*w*_(*f*) – were computed from the epoch as follows:

$${\mu }_{w}(f)=\frac{1}{|T|}\sum _{t\in T}{S}_{w}(t,f)$$

2 $$\begin{array}{c}{\sigma }_{w}^{2}(f)=\frac{1}{|T|}\sum _{t\in T}\,{({S}_{w}(t,f)-{\mu }_{w}(f))}^{2}\end{array}$$

The z-scored, trial-averaged spectrogram is given by *S*(*t*, *f*):

3 $$\begin{array}{c}S(t,f)=\frac{1}{|W|}\sum _{w\in W}(\frac{{S}_{w}(t,f)-{\mu }_{w}(f)}{{\sigma }_{w}(f)})\end{array}$$

Due to the z-score normalization, *S*(*t*, *f*) can be negative or positive.

The trial-averaged spectrograms of active electrodes revealed biomarkers of cognitive processing: an increase in the power of the high-gamma activities during word presentation, accompanied by a decrease in the low-frequency band power^34,35^. In contrast, the trial-averaged spectrograms of inactive electrodes did not show these spectral power changes (Fig. 1). High-gamma activity has previously been shown to be a biomarker of neuronal firing in human and nonhuman primate iEEG recordings^36–38^ and to correlate with the BOLD signal^39^. Low-frequency oscillations such as the theta rhythm, unlike the more focal high-gamma synchronizations of local neuronal assemblies, are thought to reflect temporal coordination of network activity across larger populations of neurons^12^.

To further understand the temporal relationship between spectral power and the frequency bands, we treated the average power as a function of time in various frequency ranges and defined them as *mean band power change*. Here, averaging of the spectrogram was performed over seven physiologically relevant frequency ranges: *low theta* (2–5 Hz), *high theta* (6–9 Hz), *alpha* (10–15 Hz), *beta* (16–25 Hz), *low gamma* (35–55 Hz), *high gamma 1* (65–89 Hz), and *high gamma 2* (90–115 Hz). The high-gamma band was split into two ranges, *high gamma 1* and *high gamma 2*, to detect any possible differences between the lower and higher ranges of these gamma frequencies and to enable calculation of one of the metrics (see “gamma consistency” below). Figure 1 shows the mean band power change in various frequency ranges for the corresponding electrodes. The stimulus-induced patterns observed in the trial-averaged spectrograms are summarized in the mean band power change plots. The mean band power change in the low frequencies is typically high before and low during a word presentation (Fig. 1b), while the mean band power change in the high frequencies is typically low before and high during the word presentation (again Fig. 1b). The corresponding panels for an inactive electrode’s mean band power change do not show those task-induced changes in the spectral power (Fig. 1c).

The mean band power change for a frequency range FB is

4 $$\begin{array}{c}{\bar{S}}_{FB}(t)=\frac{1}{|FB|}\sum _{f\in FB}S(t,f),\,t\in T\end{array}$$

The frequency ranges discussed above are denoted as follows: low-theta θ_1_ = [2, 5] Hz, high-theta θ_h_ = [6, 9] Hz, alpha α = [10, 15] Hz, beta β = [16, 25] Hz, low gamma γ_1_ = [36, 55] Hz, high gamma γ_h_ = [65, 115] Hz, high gamma 1 ${{\rm{\gamma }}}_{{\rm{h}}}^{1}$ = [65, 89] Hz, and high gamma 2 ${{\rm{\gamma }}}_{{\rm{h}}}^{2}$ = [90, 115] Hz.

### Definitions of the metrics used to identify active electrodes

#### Induced power

**Figure 2:**
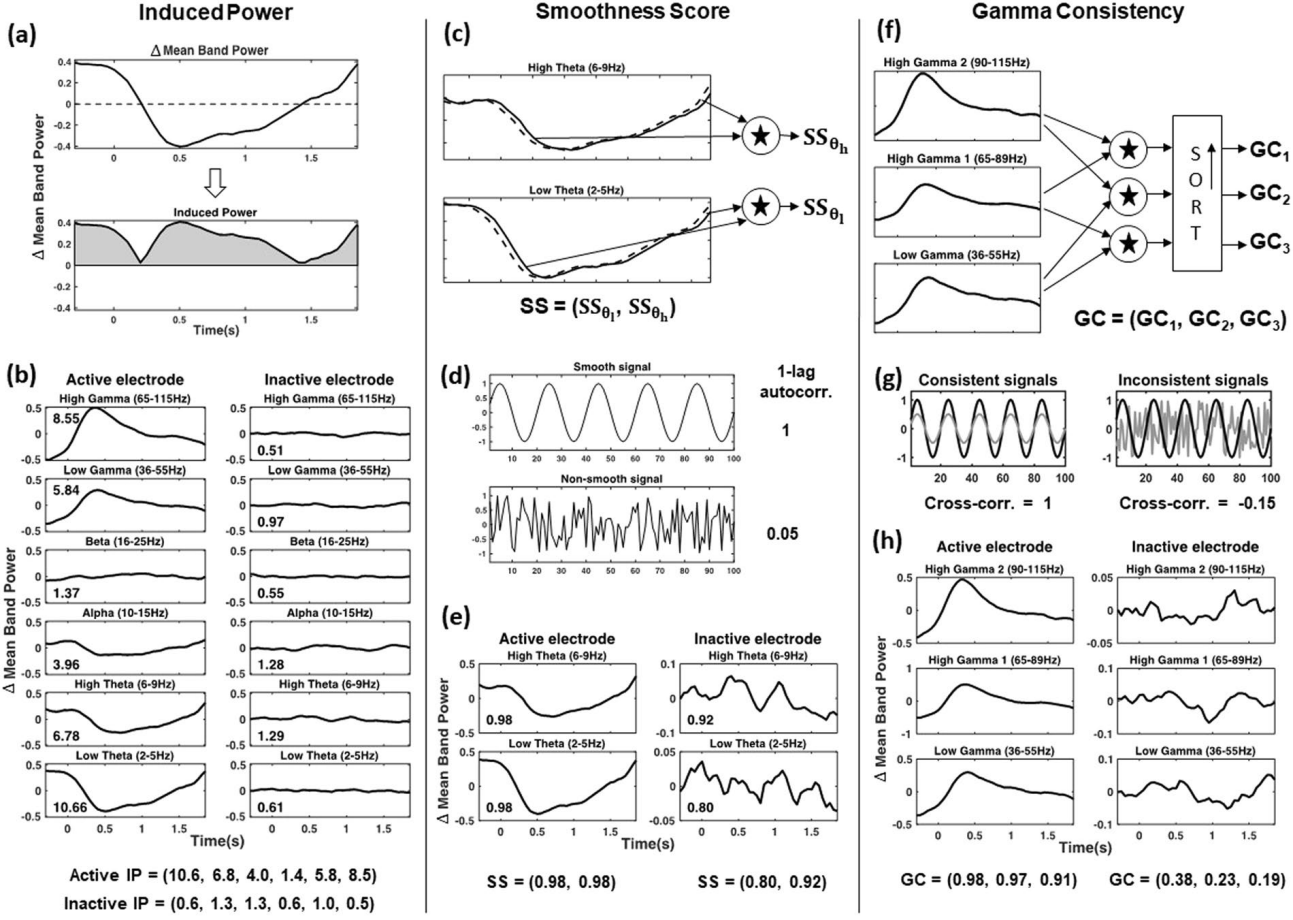
Three metrics describe the spectral power changes induced in the tasks. The figure is divided into three columns, each for one metric. (**a**) Calculation of Induced Power (IP) from the mean band power change, which is represented in the area under the absolute mean band power change curve and obtained by summing the absolute values across time. (**b**) IP values for example active and inactive electrodes are presented as the plots for the mean band power change. Notice the IP values within the plots and in the summary below the plots. (**c**) Smoothness Score (SS) is calculated with one lag autocorrelation of the mean band power change in the low-theta (${{\rm{SS}}}_{{{\rm{\theta }}}_{{\rm{l}}}}$) and high-theta bands (${{\rm{SS}}}_{{{\rm{\theta }}}_{{\rm{h}}}}$). SS is the ordered sequence of elements (${{\rm{SS}}}_{{{\rm{\theta }}}_{{\rm{l}}}}$, ${{\rm{SS}}}_{{{\rm{\theta }}}_{{\rm{h}}}}$). (**d**) Two artificially generated examples of a smooth (sine) and a non-smooth (random uncorrelated Gaussian noise) signal with the corresponding 1-lag autocorrelation values. (**e**) Smoothness scores for the mean band power change of the low-theta band and high-theta band are shown for example active and inactive electrodes quantified with the autocorrelation values. (**f**) Gamma Consistency (GC) is calculated for an example electrode as pairwise absolute cross-correlation between the mean band power changes of the three gamma frequency ranges (low gamma, high gamma 1, and high gamma 2). The pairwise absolute cross-correlation values are sorted in descending order to get three features: GC_1_, GC_2_, and GC_3_. GC is the ordered sequence of elements (GC_1_, GC_2_, GC_3_). (**g**) Two artificially generated examples of pairs of signals and their cross-correlation values. The consistent pair has two in-phase sine waves, while the inconsistent pair has a sine wave and random uncorrelated Gaussian noise. (**h**) Mean band power change in the gamma ranges is presented for example active (on the left) and inactive (on the right) electrodes with the corresponding GC values. Notice the greater synchrony between the signals of the mean band power change for an active electrode compared to an inactive electrode. Subfigures (**d**,**h**) provide a quick reference to visually assess how the range of values obtained for a given metric compare to the artificial examples.

In the z-score normalized signal of the mean band power change, task-related changes in spectral activity manifest as increases or decreases in power compared to the mean activity within a given epoch of word presentation. Thus, the absolute value of the spectral power (in the normalized signal), and consequently of the mean band power change, would be higher for active electrodes and can be used to differentiate them from inactive electrodes. For instance, the range of the mean band power change is larger in Fig. 1b than in the corresponding panels in Fig. 1c. The power does not change abruptly, i.e., it changes slowly in its local temporal neighborhood. The latency of the induced power increase depends on anatomical localization of an electrode in the brain^4,34,35^. To capture those characteristics of the mean band power change, we determined the Induced Power (IP) metric in each frequency band. It represents the sum of the absolute mean band power change across time in a given frequency band. IP was calculated separately for the six physiological frequency bands, from low theta to high gamma, as specified earlier (Fig. 2).

Using the above notation, we define Induced Power as:

$$I{P}_{FB}=\sum _{t\in T}\,|{\bar{S}}_{FB}(t)|,\,FB\in \{{\theta }_{l},\,{\theta }_{h},\,\alpha ,\,\beta ,\,{\gamma }_{l},\,{\gamma }_{h}\}$$

5 $$\begin{array}{c}IP=(I{P}_{{\theta }_{l}},I{P}_{{\theta }_{h}},I{P}_{\alpha },I{P}_{\beta },I{P}_{{\gamma }_{l}},I{P}_{{\gamma }_{h}})\end{array}$$

#### Smoothness score

For active electrodes, the mean band power change is predicted to show consistent changes across the time of word presentation in subsequent trials and, therefore, reveal smooth variation over time. Moreover, that smoothness is expected to be higher for the slower oscillations in the theta frequencies than for the faster activities in the higher gamma frequency bands. The Smoothness Score (SS) quantifies the temporal smoothness of the mean band power change. Since the smoothness of a signal can be measured by its 1-lag autocorrelation^40^, we defined SS as the 1-lag autocorrelation of the mean band power change of the low-theta band and the high-theta band. Its value lies between [−1, 1] (Fig. 2).

Hence, the Smoothness Score is defined as

$$S{S}_{FB}=Corr({\bar{S}}_{FB}(t),{\bar{S}}_{FB}(t-1)),\,FB\in \{{\theta }_{l},{\theta }_{h}\}$$

6 $$\begin{array}{c}SS=(S{S}_{{\theta }_{l}},S{S}_{{\theta }_{h}})\end{array}$$

#### Gamma consistency

Cognitive task-related changes in gamma band power tend to span a wide range of high frequencies^34,41^. Such changes can happen at the same time in multiple bands. Therefore, the mean band power change is expected to be coordinated between the gamma frequency ranges defined above (low gamma, high gamma 1, and high gamma 2) for any electrode that is active in the tasks. On the other hand, gamma activity in the particular frequency bands sampled from an inactive electrode may not necessarily demonstrate a similar coordination (Fig. 2g). In other words, the mean band power change is predicted to be more “consistent” across the gamma bands sampled from active electrodes. That motivated the use of Gamma Consistency (GC) as a metric to differentiate between active and inactive electrodes. GC is defined as the pair-wise absolute cross-correlation in the mean band power change between low gamma, high gamma 1, and high gamma 2. Since there are three possible pairs, we get three values for the absolute cross-correlation: one for each pair. The three values are sorted in descending order and denoted by GC_1_, GC_2_, and GC_3_, respectively. All GC values lie between [0, 1] (Fig. 2f). For brevity, we use the notation

7 $$\begin{array}{c}Corr(F{B}_{1},F{B}_{2})=Corr({\bar{S}}_{F{B}_{1}}(t),{\bar{S}}_{F{B}_{2}}(t)),F{B}_{1},F{B}_{2}\in \{{\gamma }_{l},{\gamma }_{h}^{1},{\gamma }_{h}^{2}\}.\end{array}$$

Hence, Gamma Consistency is defined as

8 $$\begin{array}{c}GC=(G{C}_{1},G{C}_{2},G{C}_{3})=\,{\rm{SORT}}(|Corr\,({\gamma }_{l},{\gamma }_{h}^{1})|,\,|Corr({\gamma }_{l},{\gamma }_{h}^{2})|,\,|Corr({\gamma }_{h}^{1},{\gamma }_{h}^{2})|)\end{array}$$

### Classification of active electrodes with unsupervised clustering of the metrics

We used the metrics described above to develop a method for classifying active electrodes without a need for ground truth data. Metric values were estimated for every electrode in a subject. Although the metrics for active electrode selection can be used in a subject-specific manner, to be generally applicable, they should reflect certain characteristics of electrodes that are invariant across subjects performing the same cognitive task. Therefore, we consider all electrodes pooled from the entire subject dataset. Comparison across electrodes and subjects is possible since we are looking at z-score normalized power and not the absolute power values of iEEG. Active and inactive electrodes would be expected to have different values of a given metric and tend to cluster together into two separate groups. Therefore, we performed unsupervised clustering on each metric value for all electrodes. We chose a Gaussian mixture model (GMM) with two components for clustering, instead of k-means clustering, because different numbers of electrodes may belong to the cluster of active electrodes and the cluster of inactive electrodes. The number of clusters was set to two because we expect one for each electrode type. In the study, the Gaussian distribution with the smaller mixture component was chosen as the one corresponding to active electrodes^25^, based on the results of our previous studies^3,4^.

### Evaluation of the classification approach

To evaluate the performance of the above-defined metrics, we compared the electrodes classified as active by GMM clustering with the *ground truth*: a set of electrodes labeled as active in the same verbal memory task datasets through a semiautomatic approach that involved manual inspection of the identified active electrodes^4^. In brief, the semiautomatic method had two steps based on the trial-averaged spectral power in the high-gamma band estimated for each electrode. In the first step, electrodes that had a standard deviation in the mean band power change signal of more than 0.05 across time in the high-gamma band were selected. We refer to this initial filtering as the *thresholding step*. Only electrodes that were identified in the thresholding step were considered for the second step, in which those that showed increased power in response to word presentation, as opposed to decreased power or a mixed response, were manually selected via an expert visual inspection of the mean band power change profile. The “ground truth” set was selected from 11,869 electrodes, of which 661 were identified as active. Sensitivity, specificity, and area under the ROC curve (AUC) were used to assess classification performance because of the imbalance between the numbers of active and inactive electrodes in the “ground truth.”

All the defined metrics provided multiple values (*features*) for assessing each electrode. IP contributed six features, SS contributed two, and GC contributed three. The discriminative abilities of the different features, metrics, and combinations of metrics may be different. As a result, we evaluated the classification performance for the following settings: (i) each feature individually, (ii) a group of features belonging to the same metric taken together, and (iii) all metrics combined. To understand the contributions of different metrics to the performance when all metrics were combined, we applied principal component analysis (PCA) on the correlation matrix of all metrics combined and evaluated the active electrode identification performance of each principal component.

It is worth noting that we performed clustering separately for each of the above settings and that only the feature, metric, or principal component being evaluated was used for clustering. For each setting, the sensitivity and specificity in identifying the ground truth were evaluated for 100 runs of the GMM clustering algorithm to check the robustness of the clusters. Average sensitivities and specificities were obtained for comparison. We also compared the performance of our method with that of the thresholding step of the semiautomatic approach to check whether the metrics provide any additional advantage.

## Results

**Figure 3:**
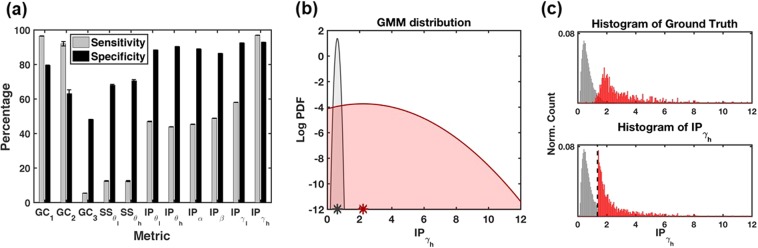
Performance of specific spectral features in identifying active electrodes. (**a**) Mean sensitivity and specificity of individual features in identifying the “ground truth” active electrodes were calculated from over 100 runs of the GMM clustering for the following features: (i) three features from the gamma consistency metric, (ii) two features corresponding to the smoothness score, and (iii) six features corresponding to the induced power metric. (**b**) Distribution of the Gaussian mixture model components found for the $I{P}_{{\gamma }_{h}}$ feature scaled with their respective component proportion and plotted as a log pdf with the means of the distributions marked with asterisks on the X-axis. Red corresponds to active and grey to inactive electrodes. (**c**) Histograms of the “ground truth” active and inactive electrodes (top panel), and of the identified active and inactive electrodes (bottom panel), are presented for the $I{P}_{{\gamma }_{h}}$ feature in grey and red colors, respectively. The dashed line indicates the threshold for choosing active electrode.

We assessed the performance of individual features (*IP* for each band, *SS* for the two theta bands, and GC features for the two gamma bands) by using sensitivity and specificity in identifying active electrodes (Fig. 3a) with reference to the “ground truth” set of electrodes established in our previous study via expert manual review^4^. The specificity, i.e., the percentage of electrodes correctly identified as inactive, was higher than 48% for all the features, with the highest specificity observed at 92.9% for the induced high-gamma power $({{\rm{IP}}}_{{{\rm{\gamma }}}_{{\rm{h}}}})$, followed by the low-gamma feature (IP_γ1_) at 92.5%. The specificity was greater than 85% for all the features that corresponded to the induced power. The sensitivity, i.e., the percentage of electrodes correctly identified as active, had a larger variation across features. All features with high sensitivity corresponded to the gamma bands (GC_1_, GC_2_, and ${{\rm{IP}}}_{{{\rm{\gamma }}}_{{\rm{h}}}}$), with the highest observed for ${{\rm{IP}}}_{{{\rm{\gamma }}}_{{\rm{h}}}}$. The sensitivity was less than 60% for the other features, with the lowest value at 5.4% for GC_3_.

In the same way, we evaluated the performance of the three metrics, and a combination of all metrics (Table 1), comparing them with the two best features identified (${{\rm{IP}}}_{{{\rm{\gamma }}}_{{\rm{h}}}}$ and GC_1_) and the thresholding step, in which electrodes with a standard deviation of more than 0.05 across time in the mean power in the high-gamma band were identified. We observed the best sensitivity for the thresholding step method, followed by the ${{\rm{IP}}}_{{{\rm{\gamma }}}_{{\rm{h}}}}$ feature. GC, IP, and all metrics combined achieved a sensitivity of more than 85%, with SS being the least sensitive. The thresholding step was expected to result in the highest sensitivity, given the way the “ground truth” set of active electrodes was identified^4^. However, we did not expect the specificity of this method to be the lowest (47.2%); the fact that it was suggests that there were many false positives in our original report that had to be manually removed in the expert manual review. The highest specificity was achieved by induced high-gamma power (${{\rm{IP}}}_{{{\rm{\gamma }}}_{{\rm{h}}}}$).

**Table 1:** Summary of the active electrode classification performance.

	GC	GC_1_	SS	IP	${{\rm{IP}}}_{{{\rm{\gamma }}}_{{\rm{h}}}}$	All	Thresholding step
Sensitivity	87.83 (4.17)	94.60 (13.48)	16.90 (1.57)	93.39 (0.14)	96.97 (0.01)	93.19 (0.01)	**100**
Specificity	65.57 (7.28)	79.36 (1.78)	60.51 (6.78)	84.48 (0.06)	**92.93 (0.02)**	83.36 (0.02)	47.15
AUC	0.855 (0.02)	0.909 (0.123)	0.362 (0.080)	0.945 (0.001)	**0.979 (0.001)**	0.938 (0.001)	0.923

Classification performance of various features, metrics and combination of metrics for finding active electrodes are provided. They are also compared with the “thresholding step”^4^ used in identifying the “ground truth” active electrodes. The thresholding step involved finding electrodes for which the standard deviation of the high-gamma power was greater than 0.05. The mean sensitivity, specificity and AUC values are averaged over 100 runs of GMM clustering and provided with a standard deviation (in parentheses). List of abbreviations: GC = Gamma Consistency metric; GC_1_ = first feature of GC metric; SS = Smoothness Score metric; IP = Induced Power metric; ${{\rm{IP}}}_{{{\rm{\gamma }}}_{{\rm{h}}}}$= induced power in high-gamma feature; and All = all metrics combined.

**Figure 4:**
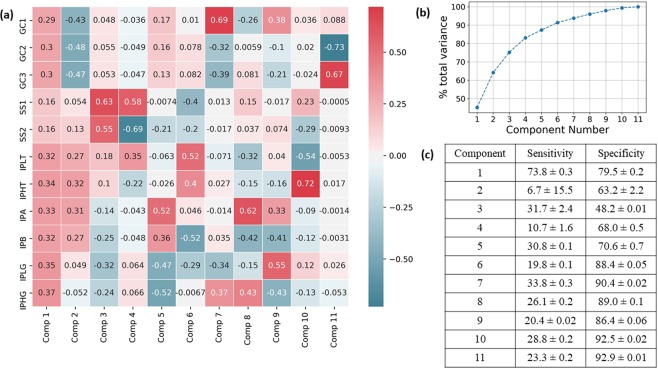
Principal component analysis of “All” metrics combined. (**a**) Principal components with the corresponding contribution of each feature. (**b**) Total variance as a function of the number of principal components used. (**c**) Mean sensitivity and specificity in identifying the “ground truth” active electrodes for each principal component.

PCA, applied to the correlation matrix of all metrics combined, revealed that the first principal component contributed 45.2% of the total variance in the data, and at least 4 components were required to explain 80% of the total variance (Fig. 4b). The contribution to the first principal component is highest for IP features, followed by GC, followed by SS (Fig. 4a). The sensitivity of the active electrode identification performance for the first principal component, 73.8%, is the highest among all components. (The sensitivities of other components are less than 35%; see Fig. 4c.)

To evaluate performance of the proposed method against other commonly used automated methods, we performed a leave-one-subject-out cross validation. In each iteration of the cross-validation, training was done on 114 subjects and tested on the remaining one subject. This was repeated for every subject. We compared the results with two widely used machine learning methods – Linear Discriminant Analysis (LDA) and linear Support Vector Machine (SVM). We used mean band power change in the high gamma band as input to LDA and SVM since the ${{\rm{IP}}}_{{{\rm{\gamma }}}_{{\rm{h}}}}$ feature achieved the best performance with our method. Our proposed method outperformed the LDA and SVM methods, achieving a higher sensitivity (Table 2). Despite a smaller specificity, our method showed considerably greater AUC scores. Leave-one-subject-out cross validation also enabled us to evaluate subject level performance of the proposed method. We observed that 97% of the subjects achieved an AUC of more than 0.90. In conclusion, our method was better in detecting more active electrodes in any one subject, presumably among the ones at the boundary between the two clusters.

**Table 2:** Leave-one-subject-out cross-validation performance.

	${{\rm{IP}}}_{{{\rm{\gamma }}}_{{\rm{h}}}}$	LDA	SVM
Sensitivity	**97.1 (12.5)**	57.4 (39.3)	48.4 (45.9)
Specificity	93.1 (7.7)	99.1 (2.4)	**99.8 (1.0)**
AUC	**0.974 (0.125)**	0.752 (0.250)	0.741 (0.281)

Performance is given for the proposed method with ${{\rm{IP}}}_{{{\rm{\gamma }}}_{{\rm{h}}}}$ feature, Linear Discriminant Analysis (LDA), and Support Vector Machine (SVM) classifier.

We performed further analysis of the GMM clusters for the best-performing feature identified, ${{\rm{IP}}}_{{{\rm{\gamma }}}_{{\rm{h}}}}$. The mixture proportion of the active electrodes cluster was found to be 14.90% (Fig. 3b). The cluster was found to have a higher mean than the inactive electrode cluster, suggesting that active electrodes tend to have higher induced power in the high-gamma band. The range of values taken by electrodes chosen as active by the ${{\rm{IP}}}_{{{\rm{\gamma }}}_{{\rm{h}}}}$ feature match the range of values of the “ground truth” set of active electrodes, which corroborates the high sensitivity and specificity scores obtained for that feature (Fig. 3c).

**Figure 5:**
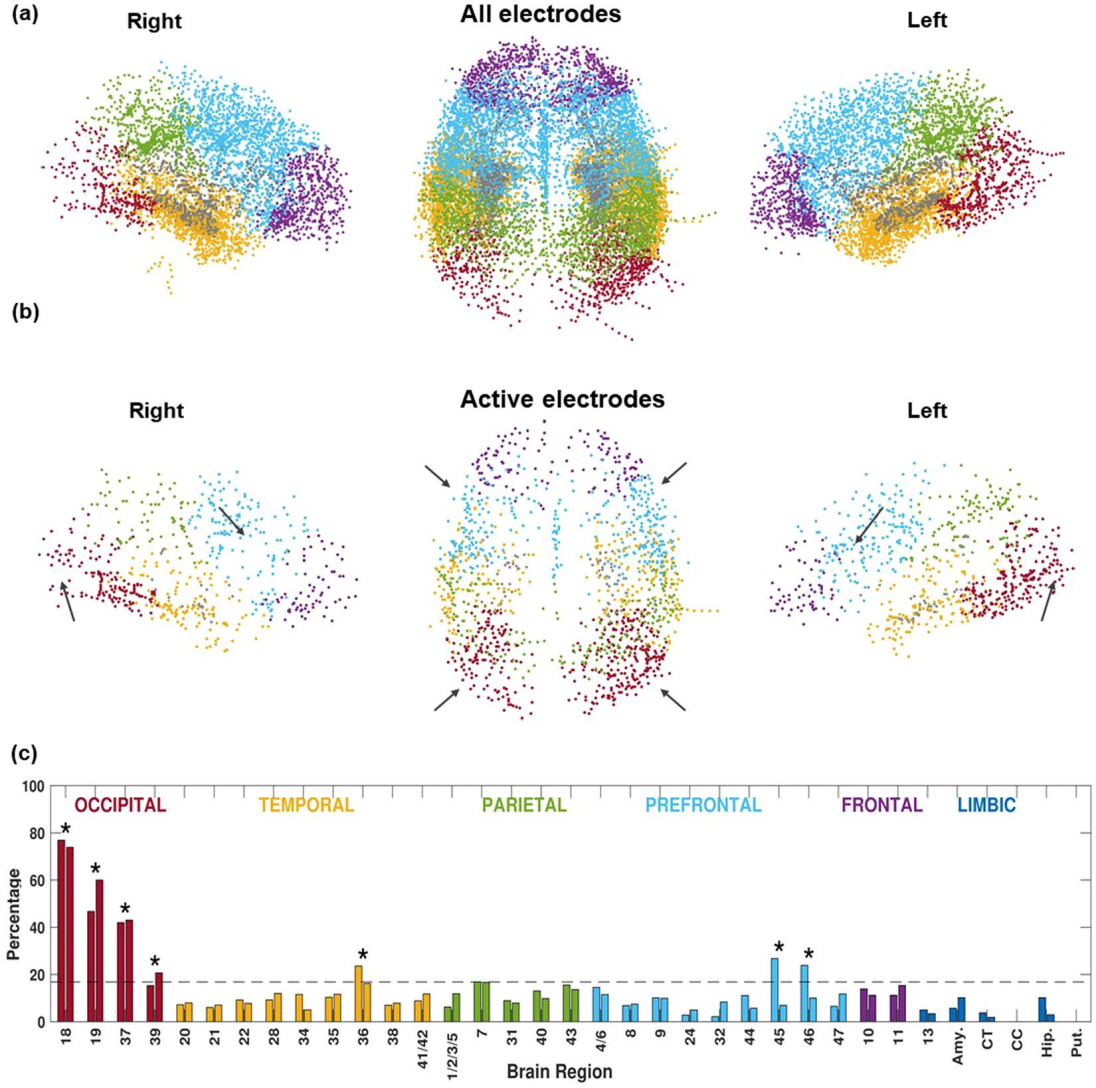
Anatomical distribution across 70 brain regions from both hemispheres reveals brain regions with the highest densities of classified active electrodes. (**a**) The locations of all implanted electrodes in the study. (**b**) The subset of those electrodes identified as active by the ${{\rm{IP}}}_{{{\rm{\gamma }}}_{{\rm{h}}}}$ feature. Hotspots with relatively high densities of classified active electrodes can be seen in the left ventrolateral prefrontal cortex (Broca’s area) and in the left occipito-temporal cortex junction, in contrast with the corresponding locations in the right hemisphere (indicated by arrows). (**c**) The proportion of active electrodes identified based on the induced power in high gamma is quantified as a percentage of all electrodes localized in each Brodmann area brain region. (The left- and right-side bars correspond to the left and right hemispheres, respectively.) The regions marked with asterisks showed more than 16.8% of the electrodes active in at least one hemisphere (85^th^ percentile). Brain regions are color-coded according to the cortical lobe: red = occipital lobe; yellow = temporal lobe; green = parietal lobe; light blue = prefrontal lobe; purple = frontal lobe; grey = all, including the Brodmann area 13, amygdala [Amy.], caudate tail [CT], corpus callosum [CC], hippocampus [Hip.], and putamen [Put.].

Finally, we validated our fully automated, unsupervised classification method by investigating the anatomical distribution of the identified active electrodes to test whether it was significantly uneven and preferentially localized to the Brodmann area (BA) brain regions implicated in verbal memory processing. The analysis was performed with the ${{\rm{IP}}}_{{{\rm{\gamma }}}_{{\rm{h}}}}\,$feature because it had the highest specificity and sensitivity scores. Only regions with at least 40 electrodes implanted (in both hemispheres combined) were included in the analysis in order to clarify data presentation. The percentage of electrodes identified as active out of the total number of electrodes implanted in any region was used to assess the effect of brain region on the distribution of active electrodes. We observed that proportions of active electrodes were significantly different across brain regions (ANOVA, *p* < 0.001, F = 25.82, n = 35 brain regions), with less than 20% of the electrodes active in most of the regions (Fig. 5c). Next, we identified the regions that showed the highest proportions in the 85^th^ percentile or above, i.e., 16.8% or more of electrodes were active. Those regions were localized in the visual cortex areas of the left and right occipital lobes (BA-18, BA-19, BA-37, BA-39), in the BA-36 of the left mesial temporal lobe, and in the BA-45 and BA-46 of the left frontal lobe. We also observed a higher density of active electrodes in the BA-45 and BA-46 of the left hemisphere (26.8% and 23.9%, respectively) compared to the right hemisphere (6.9% and 10%, respectively), which overlapped with the Broca’s area of the language-dominant hemisphere (Fig. 5b). That finding provided additional validation of our method for identifying the electrodes that are active during a verbal memory task by revealing the brain regions implicated in processing of visual and multimodal information about verbal stimuli.

## Discussion

In the era of high-density, multichannel neurotechnologies for brain recording and stimulation^32,42^, classification of active electrodes is critical for studying the physiological processes that underlie memory, cognition, and other brain functions, and for developing clinical applications. Even so, methodologies for automated identification of active electrodes have been underexplored. The majority of the prior work on identification of active electrodes, also called *channel selection*, was limited to scalp EEG^25^. Active electrode identification has been less documented for intracranial EEG (iEEG) and has typically been done with manual or semiautomatic selection of a subset of channels with meaningful signals, as exemplified in several previous studies^3,4,9,28^. Manual identification of active electrodes poses several challenges. Identification of active electrodes for individual subjects is cumbersome and subjective. Different human experts may not identify identical sets of active electrodes. The signal-to-noise ratio of the neural activity in the tasks and other subject-specific factors relating to task performance pose major challenges for manual electrode selection. For example, we observed a low specificity score for the thresholding step used in the “ground truth” method for electrode selection, showing that the currently employed methods rely on visual inspection and thus employ subjective selection criteria. For example, we identified two similar electrodes that were differentially classified by the expert reviewer as active and inactive, even though both were classified as active by our method (see Fig. S1). Furthermore, the specific set of active electrodes is prone to change over time because of shifts in electrode localization due to electrode displacement on the brain surface. Therefore, variable sets of electrodes may record from a task-activated brain region and thus qualify as active electrodes at different points in time. These issue challenge manual expert review approaches and motivate the need for automated methods for active electrode identification.

In this paper, we have presented a method that performs unsupervised classification of the data to select active electrodes in a fully automated fashion by (1) using novel metrics based on iEEG spectral content, (2) selecting electrodes from multiple subjects together, and (3) employing binary clustering to separate active and inactive channels. We found that it was possible to perform (2) by leveraging certain spectral characteristics that are common across subjects but may be difficult to discern within a single subject. Our fully automated machine-learning approach provides a means for dynamic re-identification of active electrodes across multiple features and can be used across various timepoints. The method takes advantage of basic spectral differences between active and inactive electrodes by clustering them in the space of the three metrics that we studied here. Thus, we provide a general framework for active electrode classification using spectral metrics that are easy to validate and interpret. Since the metrics were derived from basic and intuitive properties of the iEEG signals, the clusters identified in the metric space enable clear physiological validation and interpretation of the selected active electrodes in terms of brain anatomy and processes recruited in a given cognitive task. By evaluating over 10,000 electrodes recorded in over 100 subjects while they performed free recall verbal memory tasks, we have provided a systematic assessment of our proposed methodological framework.

Trial-averaged spectrograms of signals sampled from an active electrode reveal a set of known biomarkers of cognitive processing, such as increased gamma band power or suppression of low-frequency oscillations induced by stimulus presentation. Quantifiable metrics of such biomarkers can be used to separate active and inactive electrodes. Therefore, based on the biomarkers and observations of mean band power changes (Fig. 1), we proposed the IP, SS, and GC metrics to aid in automatic classification of active electrodes. Each of the above metrics is multidimensional, with each dimension called a *feature*. Since the metrics (and features) were calculated with normalized iEEG data, the features can be compared and utilized across different subjects or even tasks. Hence, they can easily be applied to any other machine-learning-based approach for classification and assessed with the standard receiver operating characteristic measures of sensitivity and specificity.

All the features with high sensitivity in identifying ground truth active electrodes correspond to the gamma bands (GC_1_, GC_2_, and ${{\rm{IP}}}_{{{\rm{\gamma }}}_{{\rm{h}}}}$). Since the active electrodes in our previous study^4^ were picked mainly based on their activity in the high-gamma band, it is natural that the features/metrics defined on high-gamma bands have high sensitivity scores. The low sensitivity obtained for features based on the low frequencies suggests that the differences between active and inactive electrodes in those frequencies are fewer than in the high frequencies with respect to the ground truth. The performance when all metrics are combined (column “All” in Table 1) is very similar to the performance of the IP metric. Further investigation using PCA on all metrics combined revealed that the first principal component gave the maximum sensitivity of all components. The highest values in the first principal component came from the IP metric. Those observations from PCA, as well as the similarity in performance between all metrics combined and the IP metric, suggest that most of the performance when all metrics are combined is primarily driven by the features in IP rather than SS and GC.

The ${{\rm{IP}}}_{{{\rm{\gamma }}}_{{\rm{h}}}}$ feature, IP metric, and all metrics combined achieved similar sensitivity and a substantially higher specificity than the thresholding step did (Table 1). In addition, our clustering method presents advantages over other automatic approaches that employ statistical testing with p-values to choose active electrodes^8,35^. First, statistical tests are typically performed over a single feature, while clustering can be performed in multidimensional spaces, as seen in the case in which metrics were used. Therefore, clustering can potentially allow for selection of active electrodes that simultaneously satisfy multiple criteria specific to each feature under consideration. Second, clustering methods like GMM are not contingent upon assumptions about the data variance, as in t-test or ANOVA^8,35^. Overall, that suggests that in finding ground-truth active electrodes, our fully automated method, using iEEG spectral metrics to identify active electrodes, provides an advantage over the thresholding step of the semiautomatic method^4^ as well as other methods that require statistical testing and/or expert review.

Previous studies used power in the iEEG spectrum along with statistical tests to select active electrodes^7^, with an emphasis on the electrodes that showed increase in power^6,8^. Our method is able to automatically identify electrodes as active with higher induced power in the high-gamma band with no *a priori* knowledge or constraint. We found post hoc that the best-performing feature classified the active electrode cluster as the one with greater induced power in the high-gamma band. That is consistent with the phenomena seen in Fig. 1 and demonstrates how our proposed method and metrics enable physiological interpretation of the identified active electrodes. For example, increased power in the high-gamma frequencies is thought to signify synchronous neuronal firing^37,38^ that is also associated with increased blood flow to the specific regions of the brain^39^, where the identified active electrodes are being recorded. Those simple metrics provide direct physiological interpretations, in contrast to the multiple or more abstract features employed in other studies that have used iEEG signals^43,44^.

Several other studies have used anatomical localization of electrodes as a criterion for selecting active electrodes for further analyses of cognitive processing^9,28^. Our approach can objectively identify brain regions with higher densities of active electrodes. Our analysis of the percentages of electrodes chosen as active by ${{\rm{IP}}}_{{{\rm{\gamma }}}_{{\rm{h}}}}$ in different brain regions revealed that the distribution of electrodes was non-uniform across the brain regions (ANOVA, *p* < 0.001). That result is congruent with the hypothesis that different sets of regions are engaged in supporting specific brain functions. In the verbal memory tasks that we used in this study, we found that the regions in Brodmann areas (BA) BA-18, BA-19, BA-36, BA-37, BA-39, BA-45, and BA-46 were ranked highest in terms of active electrode percentage, confirming our previously reported observations^4^. All those regions are highly involved in language processing, and comprise the semantic network^29,30^. Moreover, the higher density of active electrodes in the dominant left hemisphere was found for BA-44/45, BA-46, and BA-36. That observation may have useful applications in clinical determination of language dominance but requires further investigation.

In this study, we chose a Gaussian mixture in which the smaller mixture component was the one corresponding to the active electrodes, since 10–30% of electrodes are typically found to be active^25^. The method itself is generic, and prior knowledge about the metric values for active electrodes should be used in choosing the appropriate Gaussian mixture component. We applied in our framework several features based on iEEG characteristics to identify clusters of active and inactive electrodes from predefined metrics for the Induced Power, Smoothness Score, and Gamma Consistency. Still, the framework can easily incorporate other, more sophisticated features. For example, information-theoretic criteria like mutual information have previously been used to identify active electrodes^21,45^ and can be used as features for unsupervised clustering of active electrodes. Future studies are needed to assess the performance of other metrics and features.

It remains unclear how the selection of active electrodes would affect performance in verbal memory task-related classification of, for example, trials with successful and unsuccessful recall of remembered and forgotten words based on iEEG signals. Various methods for active electrode selection have been proposed to improve task-related classification performance in other tasks, including recursive channel elimination^17,22^ and genetic algorithms^23,24^. For recursive channel elimination^17^, the authors select active electrodes by recursively training a support vector machine classifier on the features of a subset of channels and eliminate the least-contributing channel until a pre-specified number of channels remain. Yang *et al*. iteratively used a genetic algorithm and an artificial neural network for classification of outcomes using the selected electrodes to find task-related activity^24^. While those methods improved classification accuracy, they tend to be computationally expensive, prone to overfitting^25^, and require ground-truth data to be trained. One way of dealing with overfitting is to use more data during training, which can be achieved by using data from multiple subjects^22^. Whether higher classification accuracy leads to a better understanding of the underlying physiology remains to be determined. The focus of our method is on finding electrodes that satisfy criteria for simple interpretation and insight into the underlying physiological processes. We considered data from multiple subjects to improve generalization of the results and their interpretation across subjects. A logical next step for this research would be to investigate the effect of active electrode selection on task-related classification performance, i.e., iEEG-based classification of successful and unsuccessful memory trials^46^.

It is important to note that we performed clustering of active and inactive electrodes grouped from all subjects to obtain a population model and separate the two classes in each subject sample based on that model. This approach addresses several key challenges such as (i) small number of electrodes to cluster within a subject, (ii) similarity and overlap in the metric values for electrodes at the boundary between the two clusters, and (iii) substantial proportion of electrodes at the boundary between the two clusters. An additional advantage of first providing a general model from grouping all electrodes is provided for many cases of subjects with a very small or a very high proportion of active electrodes, which would present other challenges for clustering within a subject. On the other hand, grouping of electrodes from multiple subjects confers an important limitation of our proposed method related to the requirement of a large number of patients to create a robust model. Unless enough samples are provided, GMM may not give clusters that would reliably reflect the underlying population of active and inactive clusters (see Fig. S2). The minimum number of total electrodes (or subjects) required will vary based on the task, number of electrodes per subject, and anatomical coverage. Nevertheless, once a general population model with a large number of samples is obtained as in our current study with thousands of electrodes from over one hundred patients, the model can be applied to other studies with smaller number of patients for the same task. It remains to be established how generalizable the model will be across different studies with the same or even a different task.

There are other limitations to our method that need to be considered. One of them is the choice of 1-lag autocorrelation as a measure of smoothness score. 1-lag autocorrelation is affected by the sampling rate, with a higher sampling rate resulting in the signal having a higher 1-lag autocorrelation value. To tackle this challenge, we resampled all the iEEG recordings to 500 Hz. In general, signal sampling frequency is an important aspect of the proposed framework– both the Induced Power and the Smoothness Score estimates will be affected by the sampling frequency. For instance, Induced Power would possibly be higher for a signal sampled at a higher sampling frequency compared to the same signal sampled at a lower sampling frequency. Therefore, it is important to deal with the limitations of the framework related to sampling frequency of the involved recordings. Two possible ways of dealing with the limitation are (i) either using recordings sampled at the same sampling frequency given that clustering relies on the relative values of the metrics for different electrodes, or (ii) using metrics that are invariant to signal sampling frequency. All in all, our method is robust as long as it is applied with careful understanding of these limitations.

Finally, our method was developed and tested in a cognitive task with human patients, specifically in verbal tasks for free recall of word lists. We found that our metric of induced power in the high-gamma band showed the highest sensitivity and specificity performance in classifying active electrodes. Increased gamma and high-gamma band activity has been shown to play a role in visual tasks^47–49^, motor tasks^50,51^, and motor-imagery tasks^6,52^, and is thought to be a general biomarker of cognitive processing in the brain^53^. Therefore, metrics based on the gamma band power, including gamma consistency, would be useful for active electrode classification in a range of cognitive, motor, and perceptual tasks. The metric values may, however, differ across various tasks. Other large studies with similar number of subjects are needed to test generalizability of the proposed method. Despite these pending questions, our results with this new unsupervised, fully automated approach to identifying active electrophysiological channels offer efficient tools for development of neurotechnologies for brain recording and modulation.

## Supplementary information

Supplementary figures

## Data Availability

Datasets used are open-access and available on the link provided in the references^31^. MATLAB code to implement our proposed method is published and can be downloaded from the following GitHub link: https://github.com/kvsaboo/TaskActiveElectrodeIdentification.
