# Supplementary material for: Unsupervised machine-learning classification of electrophysiologically active electrodes during human cognitive task performance
Source: Sci Rep. 2019 Nov 22;9:17390. doi: 10.1038/s41598-019-53925-5 (PMC6874617; doi:10.1038/s41598-019-53925-5)
Supplement: Supplementary file 1 — Supplementary figures [file 41598_2019_53925_MOESM1_ESM.docx]

**Unsupervised machine-learning classification of electrophysiologically active electrodes during human cognitive task performance**

**Authors:** Krishnakant V. Saboo^1^, Yogatheesan Varatharajah^1^, Brent M. Berry^2,3^, Vaclav Kremen^2,3,11^, Michael R. Sperling^5^, Kathryn A. Davis^6^, Barbara C. Jobst^7^, Robert E. Gross^8^, Bradley Lega^9^, Sameer A. Sheth^10^, Gregory A. Worrell^2,3^, Ravishankar K. Iyer^1^, and Michal T. Kucewicz^2,3,4^

**Affiliations:**

1. University of Illinois, Dept. of Electrical and Computer Engineering, Urbana-Champaign IL, USA
2. Mayo Clinic, Dept. of Neurology, Rochester MN, USA
3. Mayo Clinic, Dept. of Physiology & Biomedical Engineering, Rochester MN, USA
4. Gdansk University of Technology, Faculty of Electronics, Telecommunications and Informatics, Multimedia Systems Department, Gdansk, Poland
5. Thomas Jefferson University Hospital, Dept. of Neurology, Philadelphia PA, USA
6. University of Pennsylvania Hospital, Dept. of Neurology, Philadelphia PA, USA
7. Dartmouth-Hitchcock Medical Center, Dept. of Neurology, Lebanon NH, USA
8. Emory University, Dept. of Neurosurgery, Atlanta GA, USA
9. UT Southwestern Medical Center, Dept. of Neurosurgery, Dallas TX, USA
10. Baylor College of Medicine, Dept. of Neurosurgery, Houston TX, USA
11. Czech Institute of Informatics, Robotics, and Cybernetics, Czech Technical University in Prague, Prague, Czech Republic

**Corresponding Authors:** Krishnakant Saboo ([ksaboo2@illinois.edu](mailto:ksaboo2@illinois.edu)), Michal T. Kucewicz ([Kucewicz.Michal@mayo.edu](mailto:Kucewicz.Michal@mayo.edu))

**Supplementary figures**


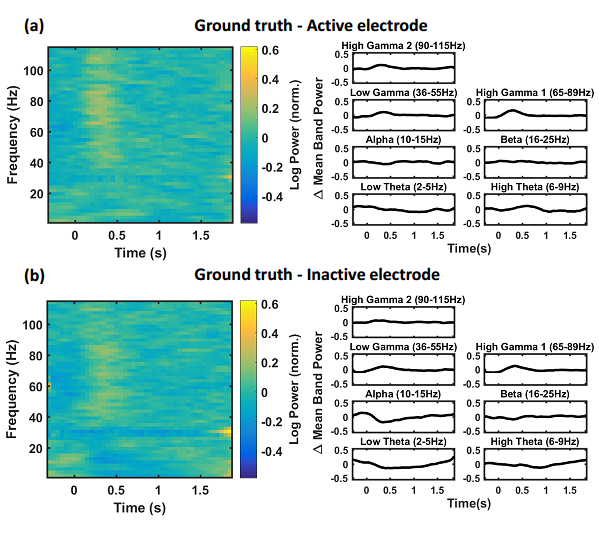


**Figure S1: Spectrograms highlighting limitations of human expert classification.** Spectrogram and mean band power for two electrodes from the same subject. (a) Electrode was identified as active in the “ground truth” expert review. (b) Electrode was identified as inactive in the same expert review. Note the similarity between the spectrograms in (a) and (b); both show a spectral profile as in Figure 1b example of an active electrode but the response magnitude is smaller. Both the electrodes in (a) and (b) were classified as active by the proposed method.

**
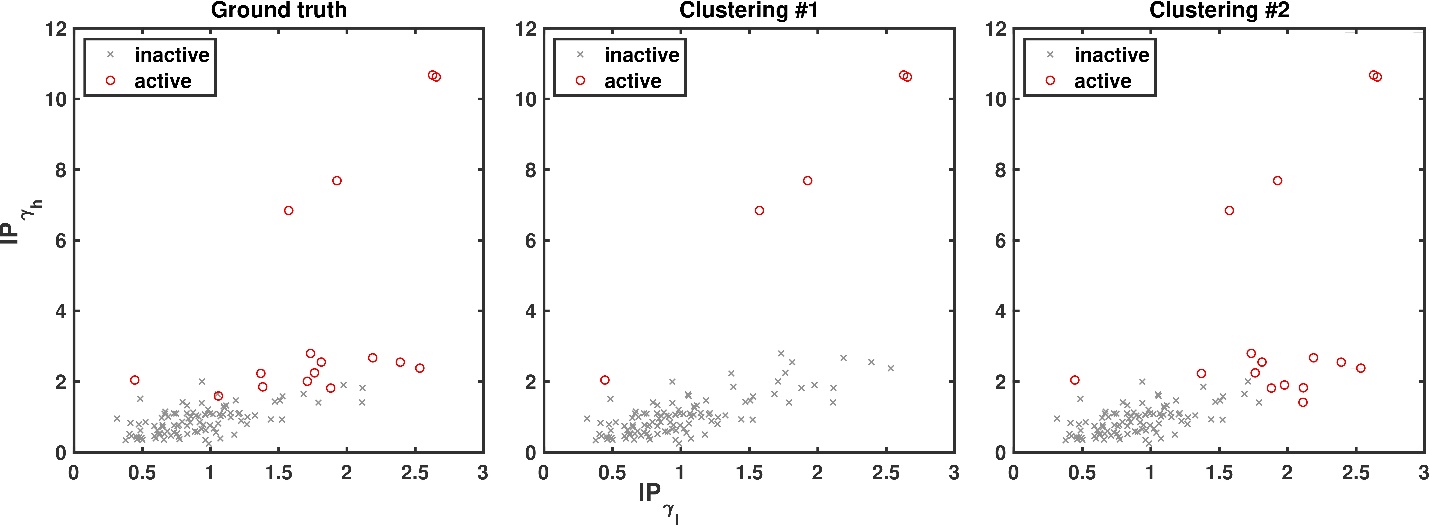
Figure S2. Per-subject clustering of active electrodes.** The electrodes of an example subject are shown in the 2D space given by features ($IP_{\gamma_{l}}, IP_{\gamma_{h}})$. (Ground truth) Ground truth active and inactive electrodes. (Clustering #1) Electrodes found as active and inactive after performing per-subject clustering in the ($IP_{\gamma_{l}}, IP_{\gamma_{h}})$ space. (Clustering #2) Electrodes found as active and inactive after performing per-subject clustering for the same subject in the ($IP_{\gamma_{l}}, IP_{\gamma_{h}})$ space but with a different random seed for clustering. Note the difference between the clusters found by the GMM due to difference in the initial seed. In per-subject clustering, only electrodes from the given subject were clustered instead of grouping electrodes from all subjects before clustering.
